# Meetings are an Important Prerequisite for Flourishing Workplace Relationships

**DOI:** 10.3390/ijerph18158092

**Published:** 2021-07-30

**Authors:** Sophie Schön Persson, Kerstin Blomqvist, Petra Nilsson Lindström

**Affiliations:** Faculty of Health Sciences, Kristianstad University, 291 88 Kristianstad, Sweden; Sophie.schon@hkr.se (S.S.P.); Kerstin.blomqvist@hkr.se (K.B.)

**Keywords:** workplace relationships, flourishing, multi-stage focus groups, old age healthcare, participatory approach, workplace health promotion

## Abstract

Relationships among colleagues, managers, and care recipients are mutually important, and need to be highlighted in workplace health promotion. The aim was to explore prerequisites for flourishing workplace relationships in a municipal healthcare setting for old people. As part of this process, we explored the staff’s suggestions on how work relationships could be improved. The study had a salutogenic and participatory approach, examining staff perceptions of what was required for flourishing relationships to be created, and their suggestions for the relationships to be more promotive. Four multi-stage focus groups, which met three times each, were conducted with staff (*n* = 26) in old age healthcare settings. A deductive analysis was performed, based on components of the flourishing concept: challenge, connectivity, autonomy, and competence. Informal and formal meetings at work were shown to build positively perceived relationships. The study describes meetings and relationships connected to the four components of flourishing. Suggestions for improving work relationships are also presented. This study contributes to workplace health promotion, and has a salutogenic and participatory focus on how to explore workplace relationships as a resource. The flourishing concept shows how workplace relationships can be explored as prerequisites for workplace health promotion.

## 1. Introduction

In recent years, there has been an increase, albeit slow, in work-related studies adopting a salutogenic approach [1,2,3,4,5], i.e., focusing on maintaining and/or increasing health-promoting resources [6,7]. The opposite is to focus on risks of illness, which is a pathogenic approach, and studies that examine risk factors and why people feel bad through, e.g., their work. A significant factor in occupational health is relationships, and research focusing on negative work-related relationships that contribute to ill health is common [8,9,10]. Therefore, there is value in conducting studies with a salutogenic approach, that in different ways and from different theories examine the workplace as a health-promoting arena to understand which factors and processes can increase sustainable work-related relationships and employees’ health [11,12,13].

Workplace health promotion focuses on factors and processes (resources) as prerequisites for doing well and feeling good at work [12]. One resource is relationships, and these could be among colleagues, managers, and, in a healthcare context, also the care recipients [3,4,14,15,16,17]. Studies about relationships at work from aspects of teamwork and communication, also in relation to a higher quality of care [18,19], are more common. However, the various interaction patterns that underlie the effects of relationships on individual health and well-being, the processes through which these effects occur [20,21], and which specific aspects of positive workplace relationships contribute to health appear to be less explored [2,15,22].

Therefore, the specific aspects of relationships at work need to be further explored. A salutogenic concept that could be used to understand which aspects contribute, in a deeper sense, to positive relationships at work, is flourishing. Flourishing, as a concept, has theoretically been described by several authors in recent years. At first, it was more individually focused and used in terms of the state a person achieves when attaining the highest levels of functioning in life and perceiving psychosocial health [23]. Seligman (2012) likewise focused on the individual’s well-being as a key to flourishing, but also identified relationships as one of the key elements. He highlighted five key elements of well-being that people need in life to be able to flourish: positive emotions, engagement, relationships, meaning in life, and accomplishment (PERMA) [24]. McCormack and Titchen (2014) confirmed that relationships are an assisting and important factor for flourishing. They argued that it is the interconnections between a person’s health experience (physical, psychical, social, spiritual), creativity, and purposeful interactions with others, that create flourishing [25].

Later, Gaffney (2015) described from a practical point of view how flourishing could be identified and developed, and presented four important components of flourishing. The first of these is challenge. A challenge can come from oneself or from another person; it may be positive or negative; and people have different approaches for meeting and managing a challenge depending on different factors in their lives. The second component is connectivity, which is about feeling connection to oneself, to one’s surroundings, and/or with other people. The third component is autonomy, which is about feeling sufficient control and having a choice about things that matter to oneself. The last component is about using one’s own competencies such as intelligence, strengths, and experiences gained from challenges. Flourishing has its foundation in the circumstances and situations that enable persons to meet challenges in life and that enable them to flourish by viewing upcoming situations with optimism [26].

A situation that many individuals find themselves in on a daily basis is the work situation. When Colbert et al. (2016) applied flourishing to a work situation, they saw that workplace relationships played a key role in promoting employee flourishing. Work relationships promoted personal growth through friendship, and the opportunity to give to and help others in a work situation. Positively experienced workplace relationships also increased job satisfaction and feelings of meaningfulness at work, supported overall life satisfaction, and had benefits for both individuals and organizations by creating flourishing [27]. However, the need to further investigate the flourishing components for employees in a workplace context has been highlighted [27,28].

From previous theoretical descriptions of the associations between flourishing and relationships [23,24,25], together with Gaffney’s (2015) practical description [26], there is both a theoretical and a practical benefit to further investigating the specific aspects of positive work relationships with flourishing as a salutogenic concept.

In summary, the introduction has highlighted that there are limited health promotion studies with a salutogenic approach concerning (a) what is required for positive workplace relationships to develop, (b) how positive workplace relationships can be enhanced, and (c) what aspects of positive relationships are already provided in work. Thus, there is a gap to fill to extend the knowledge of how workplace relationships can improve employee health and thereby also the care of care recipients.

The aim of this study was to explore prerequisites for flourishing workplace relationships in a municipal old age healthcare setting. As part of this process, we also explored the staff’s suggestions on how work relationships could be improved in their work situation.

## 2. Materials and Methods

### 2.1. Context and Participants

As the number of elderly people is increasing [29,30], it is important to be able to provide elderly care with competent staff in the future. Therefore, the current study was conducted in an elderly care context. In Swedish municipal old age care settings, nurse assistants work either on a day shift or night shift, and provide regular as well as palliative care. The managers, registered nurses (RNs), occupational therapists and physiotherapists at the residential care units usually work during the daytime on weekdays [29,30].

This study is the final part of a participatory action research (PAR) project, and was conducted in two similar general residential care units in the south of Sweden during the period 2015–2016. The selection of units for participation was made in consultation between researchers and managers. The two chosen residential care units together had about 39 residents. Altogether, four working groups, one day shift team and one night shift team from each unit, were asked to participate. The care units employed only assistant nurses, and it was those who worked in the units who were included in the study. Nurses and other care professions were employed in another department and were therefore not part of this study. All 31 assistant nurses in the four working groups were invited to participate in the study, and 26 took part. In the Results section and thereafter, we refer to the participants as “caring staff”.

### 2.2. Procedure

With our salutogenic approach, we wanted the study to be participatory and emancipatory, to give advantage to the participants in their work situation by their participation. Therefore, the study was based on methodology for PAR. A participatory approach has the intention to enable participants to extend their understanding of issues and to empower them to use their new knowledge [31]. Previous studies in this research project also investigated workplace relationships using a salutogenic approach [3,14,15]. In these studies, the meaning and significance of workplace relationships were investigated in relation to staff, managers, and care recipients. The findings showed key aspects of relationships in the workplace, see Table 1.

In the current study, we wanted to further explore how these findings could be used for a health-promoting purpose in practice. To put previous results to practical use for the participants themselves, multi-stage focus groups were formed [32]. The multi-stage focus group method is characterized by the same group exploring a question or phenomenon in depth over the course of several meetings. Letting the same group meet multiple times allows participants to get to know each other in a deeper sense. A sense of shared history develops, which distinguishes the multi-stage focus group from a normal focus group that usually meets only once. Through multiple meetings the discussions in the group can further expand and different problems can be addressed to achieve a higher level of abstraction. Multi-stage focus groups were chosen because the method itself can contribute to emancipation within the working group through ongoing discussions. Multi-stage focus groups can also be a way to create sustainable change in the workplace, as the method recognises the local context and uses the participants’ experiences. Therefore, the method was considered suitable for PAR.

During information meetings in the spring of 2015, one of the researchers (S.S.P.) verbally invited potential participants to participate, informing them about the study and handing out written information. The intention of the information meetings was to increase interest in and motivation for participating in the multi-stage focus groups. The multi-stage focus groups were conducted from September 2015 to January 2016. Each group consisted of assistant nurses from the same working groups, as the intention was to enable dialogues in which the participants would describe experiences, share thoughts, and create suggestions for how to achieve improvements. Each group met three times and between two and nine persons attended each meeting. The focus groups lasted 70–120 min and were held in meeting rooms at the participants’ workplaces. One problem with multi-stage focus groups is that it cannot be expected that all participants can attend all meetings [32]. To keep everyone updated on the process, each meeting began with a brief recap of what had been discussed last time. Those who had not attended the previous meeting were given a report of what had been discussed. However, all participants in this study attended at least two of the three meetings.

As a starting point, statements about positively experienced relationships with care recipients, colleagues, and managers were used (see Table 1). Our intention with this was to let the participants choose the subjects that were most important for them to discuss in relation to workplace relationships, rather than selecting subjects that we researchers found the most interesting. The statements were based on previous studies of positively experienced workplace relationships [3,14,15].

One researcher acted as a moderator and the other researcher (either P.N.L. or K.B.) acted as an observer. The moderator (S.S.P.) steered all the multi-stage focus group discussions by asking questions about experiences of what is required for workplace relationships to flourish, and by urging the participants to make suggestions for how to improve the workplace relationships. The observer made a written summary of the content and interaction in the group, asked for clarifications, and urged reflections in the group. Both researchers did their utmost to allow all participants to speak freely but still to guide the actual content of the discussion. At the end of each focus group, the researchers made a summary of the suggestions for improvements that had emerged, and this summary was followed by a discussion within the group about how the staff could proceed to realise their suggestions in their unit.

### 2.3. Analysis

Initially, all the researchers read the interviews several times and discussed them together. The interview material was thereafter read with a view to identifying different kinds of relationships: relationships between caring staff colleagues within the team; relationships between the day and night shift care workers; relationships between caring staff colleagues and other occupational groups such as RNs, occupational therapists, and physiotherapists; and relationships between caring staff colleagues and the manager. Upon further examination, we saw a pattern emerge, in that the specific relationships were connected to two types of meetings: informal and formal meetings. Therefore, the material was sorted into the two content areas: Informal meetings and Formal meetings.

After reading and discussing the material further, a deductive content analysis [33] was carried out, using a modified version of Gaffney’s (2015) description of the four flourishing components [26]. We related the components of Gaffney to a work situation and workplace relationship processes as follows: (1) challenges, which refers to challenging situations in everyday work that have to be faced and managed to enhance relationships; (2) connectivity, meaning concrete actions or suggestions needed for the work relationship to be mutual; (3) autonomy, which refers to the employee’s self-determination and freedom to act in work situations; and (4) competence, which relates to an expression of feeling needed and able to use personal as well as professional skills at work.

Finally, within each kind of relationship, the four key components of the concept of flourishing were applied. To identify challenges, connectivity, autonomy and competence in each relationship, the following questions were asked when examining the material: Challenges: Which relational challenges are expressed? Connectivity: Which concrete actions or suggestions are expressed, and how are the mutual relationships described? Autonomy: How is the employees’ job autonomy described? Competence: Which competence is described, and how can it be used?

### 2.4. Ethical Considerations

The written information given to the participants covered the aim of the study, the focus for the discussions in the multi-stage focus groups, and included an assurance that participation was voluntary and that data would be handled confidentially. The researchers emphasized the importance of the discussion within the focus groups remaining confidential. This information was given again at the start of each focus group.

The participants were given the opportunity to participate by their managers, but they also had the opportunity to decline without the manager knowing about it. The study was conducted in agreement with the Swedish Ethical Review Act, SFS 2003:460, and was approved by the Ethical Review Board of Lund, Sweden (dnr: 2015/565).

## 3. Results

In this section, the various workplace relationships at meetings are described and how they relate to the four components of Gaffney’s (2015) concept of flourishing [26]. Two main categories emerged in the analysis: Informal meetings as a prerequisite for flourishing and Formal meetings as a prerequisite for flourishing. The staff described their experiences of what was required for positive workplace relationships to occur, and also made suggestions for how to improve workplace relationships. These suggestions are presented at the end of each workplace relationship.

### 3.1. Informal Meetings as a Prerequisite for Flourishing

Based on the participants’ descriptions, informal meetings in daily work consisted of small talk and non-work-related conversation going on all the time between people. Informal meetings took place during work but also during breaks. Our results show that most informal meetings took place (1) between caring staff colleagues; (2) between the caring staff and the RNs; and (3) between the caring staff and the manager.

#### 3.1.1. Informal Meetings between Caring Staff Colleagues

Informal meetings between caring staff colleagues mainly occurred during breaks but sometimes also during work. Regarding the relationship between caring staff colleagues, the participants described how the challenge was to maintain good interactions with and trust between each other. Connectivity was achieved when the caring staff felt they could be open with each other and that they were able to both give and take criticism. When there was good interaction between staff, the work had more flow. Everyone took more responsibility and became more self-motivated, which contributed to increased autonomy. When the colleagues in the group knew each other, they felt more confident in work situations, as everyone’s competence was better used at work. The participants described good interaction between colleagues as a resource for high quality of care, allowing everyone to use their particular competence well.

Suggestions for quality improvement were to make time for joint breaks. The participants expressed how they longed for breaks with their colleagues as this was a time to air their views and feelings, talk about difficult work situations, and have a nice time and also talk about private matters.

#### 3.1.2. Informal Meetings between Caring Staff and Registered Nurses

Informal meetings between the caring staff and RNs occurred mainly when the RN showed up at the ward. In the relationship between caring staff and the RN, the challenge was to organize informal meetings between the two. The caring staff often perceived the RN as absent from the daily work, so that informal meetings and conversation were hard to achieve. An example of a relationship as a resource was when the RN showed up at the ward just to ask if the caring staff had any questions or support needs. Another example was when the RN was available on the phone and the caring staff felt safe to call and ask about things. Such relationships between the RN and the caring staff were considered to contribute to connectivity. With stable connectivity in the relationship, the caring staff’s autonomy was considered to increase; for instance, the caring staff would feel confident about making decisions on their own if required. When the RN involved the caring staff in decisions about care recipients, they felt that their competence in the organization was not only being used but also increased. By taking advantage of the staff’s joint knowledge about the care recipients, the RN made the caring staff feel needed.

A suggestion from the caring staff was to introduce a notebook at the ward as an easy way to note and collect questions or information to the RN without having to leave the ward to ask the RN for advice in every single case.

#### 3.1.3. Informal Meetings between Caring Staff and the Manager

Informal meetings between caring staff and the manager occurred mainly during breaks. Challenges in the relationship between the caring staff and the manager were to create mutual respect between the two, to make the manager understand the importance of being fair, and for the manager to treat everyone equally and to express their expectations clearly. The participants said that when the manager dared to be a manager with clear directives, and when expectations were clearly stated, this created connectivity between the caring staff and the manager. When the caring staff felt respected and taken seriously, and when the manager showed respect for the caring staff’s thoughts by listening and being part of the work group, autonomy was strengthened. When the manager asked the caring staff for their opinions, the participants felt that their competence was put to use in the organization and that their knowledge was considered important.

A suggestion from the caring staff was that the manager should have more open discussions with the caring staff, for instance, about planning and routines for temporary staff.

### 3.2. Formal Meetings as a Prerequisite for Flourishing

According to the participants, formal meetings had a defined time and agenda. Formal meetings were staff meetings, care planning meetings. and daily report meetings. Formal meetings were held: (1) between the caring staff colleagues; (2) between day and night shift workers; (3) between the caring staff and other occupational groups; and (4) between the caring staff and the manager.

#### 3.2.1. Formal Meetings between Caring Staff Colleagues

Formal meetings between caring staff colleagues were mainly staff meetings. One challenge was to plan formal meetings with time to talk about issues in the caring staff group’s daily work and routines. In other words, the caring staff needed their own time to talk about matters that were important to them without the manager present. To have the opportunity to discuss freely was described to contribute to connectivity. By discussing work routines and their caring activities, the participants felt that they grew as professionals together. They also said that discussions between colleagues contributed to autonomy. It was described that the “any other matters” item on the agenda at staff meetings provided an opportunity to speak as a group, which increased the caring staff’s ability to use their competence at work in a better way.

A suggestion from the participants was that they should have their own time at staff meetings, without the manager present, to be able to address issues that only concerned the caring staff.

#### 3.2.2. Formal Meetings between Day and Night Shift Caring Staff

Formal meetings between day and night shift staff were mainly care planning meetings. However, they were difficult to arrange because of different working hours and therefore were rarely scheduled. A challenge between night and day staff was to be open to each other’s different work situations. Many routines hindered an understanding of each other’s different work circumstances, but also led to an unawareness of each other’s work. Another challenge was to create a sense of belonging between the night and day shifters. Even if they were working in the same place and with the same care recipients, the participants did not really feel that they were a united team. When the overlap between night and day shift worked well and the groups sat down together to review what had happened during their shift, feelings of connectivity between the night and day staff arose. Autonomy was increased when the meeting between night and day staff provided an opportunity to talk about something that had happened and to explain, or get an understanding of, why a task (e.g., cleaning) had not been performed.

A suggestion was to arrange discussions between day and night staff to adopt common goals and missions and make everyone aware of the importance of a common approach, not only for their own wellbeing but also for the benefit of the care recipients. By discussing these issues, everyone’s competence could be strengthened. A further suggestion from the participants, regarding how the relationship could be strengthened and used as a resource, was that working hours between shifts should overlap a bit more to give a better opportunity to meet and talk to each other, now this time was experienced as too short.

#### 3.2.3. Formal Meetings between Caring Staff and Other Occupational Groups

Formal meetings between caring staff and other occupational groups largely consisted of care planning meetings. In the relationship between the caring staff group and other occupational groups such as RNs, coordinators, physiotherapists, occupational therapists, and staff at the hospital, the challenge was to create respectful verbal and also written communication. Cooperation and discussions with professionals from other occupational groups at formal meetings were found to contribute to connectivity.

Suggestions for how to improve connectivity were to have structured follow-ups and dialogues about the caring work together with other occupational groups. When these dialogues worked well, the feeling of participation and autonomy increased. The participants suggested, in addition, regular common discussions about how staff should act in difficult work situations, and clearly stated routines and values when the caring staff had to make their own decisions in challenging work situations. The participants expressed a desire to use their caring competence by cooperating with other occupational groups and believed this would be a way to improve the quality of care.

Other suggestions from the caring staff were to preserve and develop the meetings with the RN on a weekly basis, focusing on individual care recipients, and that the RN should be present in the mornings at the overlap meetings between night and day shifters.

#### 3.2.4. Formal Meetings between Caring Staff and the Manager

Formal meetings between the caring staff and the manager mainly took place at staff meetings. The challenge in formal meetings with the manager was to save meeting time during staff meetings to talk about important matters, mainly relating to how to improve the care of the care recipients. However, the participants said that much time during these meetings was spent on financial and information issues, which was not always perceived as the most important by the caring staff. Another challenge was to get the manager to stick to decisions and provide the same information to all the working groups, as they did not have staff meetings together. A manager who took the experience of the staff into consideration during discussions was regarded as contributing to connectivity between them. When they felt listened to, the participants experienced that their autonomy was strengthened. If the manager discussed their work and work situation with them, their skills could be better used in their work and they thereby felt that their competence was used.

A suggestion from the caring staff was that the manager could provide more written information instead of oral information during staff meetings. This would make more time available for important discussions between the manager and the caring staff about the care recipients and the daily caring work and aspects of the work situation.

In summary, a methodological result of the study process with multi-stage focus group meetings is that this method had an emancipatory effect, as all participating groups decided to proceed with their own suggestions and tried to put them into action at their wards. In order to give an overview on concrete examples of improvements, we condensed all the staff’s suggestions and related them to the various relationships, and informal or formal meeting type (see Table 2).

## 4. Discussion

Positive relationships characterized by challenge, connectivity, autonomy, and competence could have an enhancing effect on employees’ health and could be useful as a starting point in workplace health promotion to develop a flourishing workplace for care professionals. To achieve this, conditions need to be created for both informal and formal meetings based on the staff’s experience.

Informal and formal meetings with colleagues, other occupational groups and the manager are important for enhancing employee health. While the informal meetings in this study group were important for the ability to exchange information and make everyday decisions, the formal meetings had a predetermined agenda and gave an opportunity to discuss issues relating to the care recipients. A common factor in informal and formal meetings was the experience of support and belonging, and being able to exchange thoughts and share feelings in the different meetings. This was important, not only for job performance, but also for well-being at work. This supports previous research suggesting that a workplace atmosphere that allows open-mindedness and a positive social climate is a resource for making work comprehensive, manageable, and meaningful [2]. Furthermore, in order to create sustainable workplaces and an organization with flourishing individuals, it is important to build a work structure based on positive relationships [13,27,34,35]. To achieve this, well-thought-out strategies for informal and formal meetings in the workplace are required.

Strong relationships with colleagues and managers and positive relationships with care recipients are closely linked. Our results showed that positive informal and formal meetings seemed to have a dual purpose. They made the staff themselves flourish at their workplace, but they were also a prerequisite for providing good care and having positive relationships with care recipients, which was also central to their own well-being. In the focus groups, the caring staff rarely talked about relationships with care recipients. Instead, they spoke about relationships with colleagues, professionals from other occupations, and managers. The caring staff chose to talk about their workplace relationships, not primarily to improve their own well-being but to achieve improved relationships with care recipients as a way of providing better care, which ultimately improved their own well-being. In human care professions, when employees are faced with high emotional demands [34], awareness of types of work relationships and why they function as a resource and as a prerequisite for flourishing is valuable.

A salutogenic approach to workplace health needs to emphasise positive relationships based on respect, security, and reciprocity. One interpretation of our study results is that there existed more of a “we-feeling” between caring colleagues working the same shift and more of an “us-and them feeling” between people from different shifts and representing different care professions. The we-feeling contributed to togetherness among the caring staff and created flow and confidence at informal meetings. This “we-feeling” of togetherness also contributed to good formal meetings because of respect, security, and strong reciprocity. Such a sense of belonging and security has also been shown in previous research [3] to contribute to well-being as well as recognition of self-worth. On the other hand, the results in this study also showed more of an “us-and-them feeling” in the relationships with caring staff from other shifts, the manager, and other occupational care groups. Hence, a major part of the challenge of turning the relationships with the manager and other occupational care groups into a resource seemed to lie with the organizational structure, as physical meetings in daily situations were sometimes considered inadequate. If an organization relies on interactions between people, relationships should be emphasized as more important to the organization than, for instance, organizational principles and structures [36]. By using the relationships as a tool for the development of the organization and by having a less hierarchical structure—a structure that is based on a democratic mindset between different professions—everyone’s competence can be anchored in the overall organization and everyone can work towards the same goal [35]. This salutogenic approach may in turn benefit the flourishing of the individuals, and the organization [27,28].

### 4.1. Practical and Theoretical Implications

In this study with a salutogenic and a participatory design, practical and theoretical implications where deeply intertwined. A theoretical implication is that the concept of flourishing, and its four specific components, can be used in practice to identify, interpret, and explain positive relationships, and situations and elements of a workplace that affect employee health. The components of flourishing have links to the three components of motivational needs in Ryan and Deci’s Self-Determination Theory (2000): autonomy, competence, and belongingness [37]. These three components create inner motivation for the individual to act in situations or in relationships that are important for health experience and well-being. There is also a connection between the concept flourishing and the model of person-centredness [28]. Person-centredness is usually discussed in relation to care, but an organization can also work in a person-centred manner with its employees and in the organization of work. Different dimensions of relationships at work become important to understand and improve, as relationships are vital for flourishing. Healthful relationships among employees may thereby also contribute to a person-centredness culture.

A practical implication is to use meetings, both formal and informal, as a means to enhance relationships in everyday practice. Conditions for meetings provide the opportunity for people to share experiences and get to know each other in the work group, which in itself contributes to a health-promoting workplace [14,15,38]. Using the concept of flourishing both in the planning of work tasks and in issues concerning the psychosocial work environment, could contribute to a flourishing and sustainable workplace. Using the four components in formal meetings enables the creation of a clear model to show what challenges and suggestions have emerged, and thus makes it possible to develop connectivity, autonomy, and use of competence in an optimal way. The identified challenges and suggestions for improvements can, for instance, undergo process evaluation at the staff meetings, in order to make the relationships a resource for the flourishing of individuals and the organization alike. It is advantageous to base dialogues on the four flourishing components to highlight both the salutogenic aspects and the challenges to creating a sustainable workplace that causes the individual workers to flourish. Previous research about using dialogue as a tool has shown that a structured dialogue model makes it easier to notice what is happening in daily work relations and provides the possibility to work in a promoting way [38].

Furthermore, a practical implication of informal meetings is to create conditions for things such as joint breaks. Conditions for informal meetings contribute to the opportunity for the staff to share their thoughts and feelings about different work situations. This promotes social relationships and the opportunity to give each other more support in everyday practice. Relationships can function as a job resource that helps employees to manage strain and job demands [39]. Especially in human-related work, such as healthcare, work tasks are often challenging and energy-draining, on the one hand, and energy-giving through daily human meetings, on the other [34].

Regardless of the subject, dialogue is essential in research with a salutogenic and participatory approach. In this study the dialogues in the multi-stage focus groups developed during the study process and had the effect that all groups decided to proceed with the suggestions and try to put them into action. Our interpretation is that the groups themselves, based on the participatory approach, were an enhancing action for the workplace relationships among the healthcare staff. This further justifies dialogue as useful in improvement work to achieve a health-promoting workplace. This should be taken into account in public health and workplace health promotion, considering society’s rationalisation efforts, according to which, meetings should be minimized and must be effective and production-oriented.

### 4.2. Limitations and Trustworthiness

The methodological aspects of credibility, dependability, and transferability need to be considered for a study to be seen as trustworthy [40]. From a credibility perspective, multi-stage focus groups were considered appropriate for this study, as it was hoped they would create a reflective environment for the participants, be emancipatory, and yield detailed data. Although a situation where the participants know each other very well can lead to a lack of discussion, a sense of security within the group facilitates a trustful discussion where participants feel free to share their feelings, thoughts, and perceptions [41]. If the study is to be repeated in the future, the focus group method may need to be modified according to any current pandemic restrictions, for example, by offering digital participation or smaller group sizes. Results obtained using a deductive analysis approach can be questioned from a credibility point of view [40], as the deductive analysis brings a risk that unexpected results may be neglected. However, the participants’ reflections and suggestions were often unexpected. Among the unexpected results was the large gap in the relationship between night and day staff. We were also surprised that the relationship with care recipients was hardly discussed. At the meetings, the participants instead focused on the relationship with their colleagues since a good relationship with their colleagues and the manager was a prerequisite for good care and a positive relationship with their care recipients. Being a study with a participatory approach, the weakest part is perhaps the lack of follow-up of whether the participants’ suggestions for changes were introduced and how they turned out. This weakness was due to limited time for the project.

The researchers have nursing and public health backgrounds, which strengthens the credibility as it enhanced the possibility to see things from different perspectives. The fact that all researchers participated in the analysis and that there was high agreement between them also strengthens the study’s credibility. Similar issues were discussed in the four focus groups, which demonstrates dependability. The transferability of the results is enhanced by descriptions of the study context, the participants, and the procedure and analysis process [40].

## 5. Conclusions

In view of the increased rationalization in society, in which meetings are minimized and are required to be strictly effective and production-oriented, our results regarding the importance of informal and formal meetings and work relationships need to be taken into account if we aim to create sustainable health-promoting workplaces. This study contributes to workplace health promotion research with its salutogenic and participatory approach and represents a new opportunity to explore and understand workplace relationships as a resource by strengthening, or maintaining, positive activities and processes. It is therefore vital to develop dialogues about relationship processes, and raise awareness of the importance of different relationships for improving the work situation and healthcare. The study also shows that the components of flourishing could be used to create a model for such dialogues. Taking the employees’ own experiences into account in group dialogues seems to improve employee engagement and could be a fruitful way to conduct workplace health promotion interventions. However, further studies are required to explore the utility of our results in intervention studies, in various workplace settings, between night and day shifts, and among different occupational groups.

## Figures and Tables

**Table 1:** Examples of statements made in multi-stage focus group discussions about workplace relationships with care recipients, colleagues. and managers.

**Positively Experienced Relationships with Care Recipients**
There is a value in sharing one’s own everyday events with the care recipients. A deeper relationship allows for more trusting conversations and is important for understanding each other’s situation better. With a deeper relationship it is easier to meet and see the care recipient’s needs. Being appreciated by the care recipient makes me feel needed and makes my work feel meaningful.
**Positively Experienced Relationships with Colleagues**
I feel good about having an opportunity to air my views and feelings about matters concerning the work with my colleagues. I get a lot out of being able to feel community (trust, pride, harmony) with my colleagues. It is important that the work flows well, and that we have good cooperation and good understanding for each other. It is important that I feel respected by my colleagues.
**Positively Experienced Relationships with Managers**
I appreciate it when the manager feels like “one of the group”. It is important to me that the manager is open (e.g., dares to ask for help; is open to new suggestions and questions). It is important that the manager delegates or leaves new tasks to me, because then I develop. It is important that the manager listens to me and sees my work, because then I feel security/trust. It is important that the manager gives me encouragement and motivates me, because then I become engaged. It is important that I can see that my work is a part of a greater whole (in the organization).

**Table 2:** The two categories of meetings that lead to flourishing of different work relationships, and participants’ (condensed) suggestions for improvement work.

Categories	Improvement Suggestions
**Informal meetings:**	
Between caring staff colleagues	There should be time for joint breaks, as these allowed time for the staff to air their views and feelings about what needed to be talked about.
Between caring staff and RNs	An information notebook should be introduced at the ward as an easy way for staff to access information.
Between caring staff and the manager	The manager should coordinate with the caring staff, for instance, about planning and routines for temporary staff.
**Formal meetings:**	
Between caring staff colleagues	The caring staff should have their own time at staff meetings, without the manager present.
Between day and night shift caring staff	Working hours should overlap more, to give the opportunity for dialogue.
Between caring staff and other occupational groups Between caring staff and the manager	Meetings about the caring work should be held on a weekly basis with focus on individual care recipients, with structured follow-ups. The manager should provide more written information to give time for discussions about the care recipients and the daily work.

Note: Registered nurses (RNs).

## Data Availability

Not applicable.
